# Modeling the response of ON and OFF retinal bipolar cells during electric stimulation

**DOI:** 10.1016/j.visres.2014.12.002

**Published:** 2015-06

**Authors:** P. Werginz, H. Benav, E. Zrenner, F. Rattay

**Affiliations:** a Institute for Analysis and Scientific Computing, Vienna University of Technology, 1040 Vienna, Austria; b Center for Ophthalmology, University of Tübingen, 72076 Tübingen, Germany; c Center for Integrative Neurosciences, University of Tübingen, 72076 Tübingen, Germany

**Keywords:** RP, retinitis pigmentosa, RBC, retinal bipolar cell, CBC, cone bipolar cell, MPDA, multi-photodiode-array, FEM, finite element method, Retinal implant, Electric stimulation, Retinal bipolar cells, Compartment model

## Abstract

Retinal implants allowing blind people suffering from diseases like retinitis pigmentosa and macular degeneration to regain rudimentary vision are struggling with several obstacles. One of the main problems during external electric stimulation is the co-activation of the ON and OFF pathways which results in mutual impairment. In this study the response of ON and OFF cone retinal bipolar cells during extracellular electric stimulation from the subretinal space was examined. To gain deeper insight into the behavior of these cells sustained L-type and transient T-type calcium channels were integrated in the synaptic terminals of reconstructed 3D morphologies of ON and OFF cone bipolar cells. Intracellular calcium concentration in the synaptic regions of the model neurons was investigated as well since calcium influx is a crucial parameter for cell-to-cell activity between bipolar cells and retinal ganglion cells. It was shown that monophasic stimulation results in significant different calcium concentrations in the synaptic terminals of ON and OFF bipolar cells. Intracellular calcium increased to values up to fourfold higher in the OFF bipolar model neuron in comparison to the ON bipolar cell. Furthermore, geometric properties strongly influence the activation of bipolar cells. Monophasic, biphasic, single and repetitive pulses with similar lengths, amplitudes and polarities were applied to the two model neurons.

## Highlights

• Biphasic stimuli with opposed polarity activate ON and OFF BP cells differently.
• Synaptic [Ca]_i_ reaches levels to initiate synaptic activity during stimulation.
• Axonal length influences de- and hyperpolarization of ON and OFF BP cell compartments.

## 1 Introduction

**Fig. 1:**
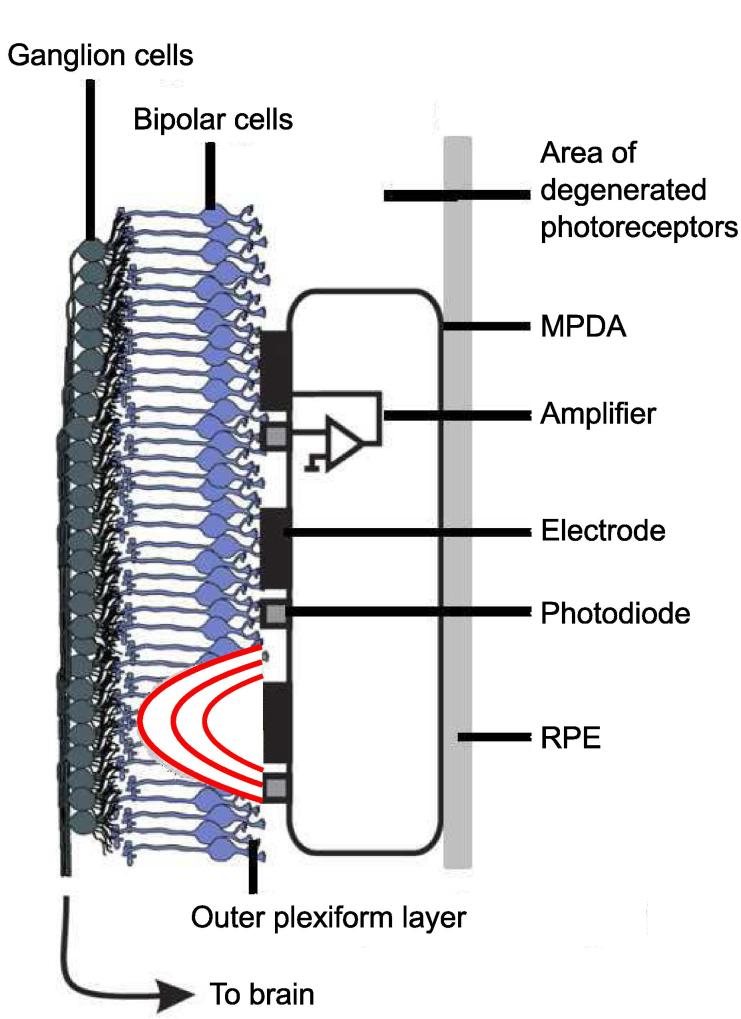
Tübingen subretinal implant and its location relative to the retina –The micro-photodiode-array (MPDA) is surgically inserted between the retinal pigment epithelium (RPE) and the bipolar cell layer into the area formerly occupied by photoreceptors. Each unit of the MPDA contains a photodiode capturing incident light, an amplifier circuit and a stimulating electrode. The light-dependent voltage generated by these electrodes primarily stimulates bipolar cells. Iso-potential lines generated by one stimulating element are shown in red. The visual information is then projected to the brain through ganglion cell axons after activation of the retinal network.

Neuroprosthetic subretinal implants for blind patients have shown that restoration of vision is principally possible (Zrenner et al., 2011). The quality of artificially created vision has not yet reached levels comparable to natural vision in humans, and this goal may be a difficult one to achieve. Subretinal implants in retinitis pigmentosa (RP) patients suffering from photoreceptor degeneration are located in the area formerly occupied by rods and cones, between the retinal pigment epithelium and the outer plexiform layer (Fig. 1).

Due to their location, retinal bipolar cells (RBC) are assumed to be the primary target of extracellular electrical stimulation through a subretinal multi-photodiode-array (MPDA). Voltage-controlled stimulating pulses create a longitudinal voltage gradient along the membrane of RBCs.

One of the factors limiting the resolution of artificially generated visual percepts may be the simultaneous and unselective stimulation of ON-type and OFF-type cone bipolar cells (CBC). These initiate the functionally opposing retinal ON- and OFF-pathways. Simultaneous activation of the two pathways could potentially result in a mutual cancellation, such that no visual perception would be elicited. While an exact annihilation is highly unlikely to be caused by a subretinal implant, there is sufficient reason to be concerned about achievable contrast and resolution. Although several experimental studies (e.g. Cai, Ren, Desai, Rizzo, & Fried, 2011; Twyford, Cai, & Fried, 2014) have introduced more sophisticated stimulating approaches such as high-frequency stimulation no appropriate stimulating strategies to avoid co-activation of the ON and OFF pathway have been found so far.

The cellular processes occurring during extracellular stimulation under in vivo conditions in retinal implant patients are largely inaccessible to presently available measurement techniques. A better understanding, especially of membrane currents and related biophysical events, is required for our ability to improve the stimulation strategies used today. With focus on RBCs, we here describe a modeling approach for deepening our understanding of these processes. Previous conductance-based models of RBC responses to electrical stimulation exist, however, these assumed no presence of voltage-gated ion channels (Resatz, 2005), used single-compartment morphologies (Gerhardt, 2010; Kameneva, Meffin, & Burkitt, 2010) or were based on lower vertebrate data (Usui, Ishihara, Kamiyama, & Ishii, 1996). This new model is based on realistic morphological, immunochemical and electrophysiological data from ON-type and OFF-type CBCs of the rat, due to the good availability of experimental rat data today and relatively simple experimental verification possibilities. Furthermore, the mammalian retina has many properties in common (Wässle, 2004), making these modeling results more applicable to human clinical studies. Two voltage gated calcium ion channels of rat CBCs were integrated into the model through fitting of Hodgkin-Huxley-like (Hodgkin & Huxley, 1952) equations for ionic currents to published electrophysiological recordings. In combination with a model which describes the change of intracellular [Ca^++^]_*i*_, this allowed us to investigate the synaptic activation of CBCs during extracellular stimulation. We can therefore make testable predictions about the influence of different stimulation paradigms on retinal activation and their suitability for sustainable, selective stimulation of retinal ON- and OFF-pathways using a subretinal implant.

## 2 Materials and methods

The calculation of transmembrane voltage V_*m*_ and ionic currents I_*ion*_ during extracellular stimulation requires quantitative knowledge on the extracellular voltage V_*e*_ which is generated by the electrodes on the MPDA in a subretinal location. Therefore, the calculation is realized in two separate and methodologically different steps. In the first step V_*e*_ is calculated and in the second step the response of a target cell is evaluated using a multi-compartment model (Rattay, 1999; Rattay et al., 2003).

### 2.1 Extracellular potential

**Fig. 2:**
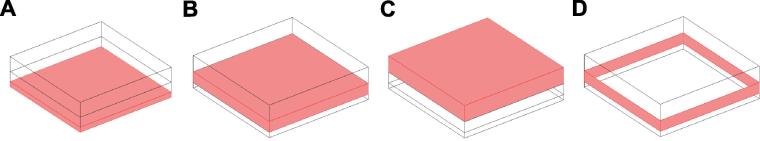
Volume used to calculate the extracellular potential generated by an electrode – Edge length is 2000 μm. (A) Chip layer, height 100 μm. (B) Retinal layer, height 300 μm. (C) Silicone tamponade, height 300 μm. (D) The outer boundaries of the retinal layer were used as current sink and had an electric potential of 0 V. To calculate the external voltage $V_{e}$ at each compartment center the potentials at given coordinates were evaluated, i.e. the morphology was virtually placed into the volume.

For the calculation of V_*e*_ a specific electrode configuration was implemented and positioned into a defined spatial volume (Fig. 2) also containing the electrode carrier or chip (2000 × 2000 × 100 μm, resistivity $\rho =10^{6}\Omega \mathrm{cm}$, Fig. 2A), a part of the retina (2000 × 2000 × 300 μm, ρ=57Ωcm (Resatz, 2005), Fig. 2) and a slice of the silicone tamponade (2000 × 2000 × 300 μm, $\rho =10^{6}\Omega \mathrm{cm}$, Fig. 2C) which replaces the vitreous body in retinal implant patients after vitrectomy (Sachs, Schanze, Brunner, Sailer, & Wiesenack, 2005). The calculation of V_*e*_ was performed with a finite element method (FEM) using commercially available software. $V_{e}$ was evaluated for each compartment center, i.e. the cell was virtually placed into the volume.

Monopolar stimulation was realized using a disk electrode with 50 μm diameter and a height of 10 μm for stimulation attached to the surface of the chip layer. The distant return electrode used in the retinal implant (Zrenner et al., 2011) was simulated by setting the boundary conditions of the retinal layer to ground at its outer boundaries (Fig. 2D).

### 2.2 Transmembrane potential

The theoretical basis for calculating the influence of V_*e*_ on V_*m*_ in a multi-compartment model with an arbitrary morphology is based on the cable equation and has been presented previously (Rattay, 1999). For the computation a single point in the center of either a cylinder or sphere electrically represents the compartment. Applying Kirchhoff’s law of charge conservation to a compartment leads to:

(1) $$I_{C}+I_{\mathrm{Ion}}+I_{R}=0$$

with $I_{C}$ denoting the capacitive current to load the membrane, $I_{\mathrm{Ion}}$ the ionic current across the membrane and $I_{R}$ the intracellular current flow to the neighbored compartments.

Considering that in the n-th compartment V_*m*,*n*_ = V_*i*,*n*_ − V_*e*,*n*_ where V_*i*_ is the intracellular voltage and V_*e*_ is the extracellular voltage referred to the compartment center, and introducing the reduced membrane potential V (Hodgkin & Huxley, 1952) with V_*n*_ = V_*i*,*n*_ − V_*e*,*n*_ − V_*r*,*n*_, where V_*r*_ is the resting membrane potential, the following system of ordinary differential equations describes the V_*e*_-dependent change of V over time for each compartment (Rattay, 1999):

(2) $$\frac{{\mathrm{dV}}_{n}}{\mathrm{dt}}=\left[-I_{\mathrm{ion}\text{,}n}+\frac{V_{n-1}-V_{n}}{R_{n-1}/2+R_{n}/2}+\frac{V_{n+1}-V_{n}}{R_{n+1}/2+R_{n}/2}+...+\frac{V_{e\text{,}n-1}-V_{e\text{,}n}}{R_{n-1}/2+R_{n}/2}+\frac{V_{e\text{,}n+1}-V_{e\text{,}n}}{R_{n+1}/2+R_{n}/2}+...\right]\frac{1}{C_{n}}$$

where *t* is the time in ms, R_*n*_/2 represents the internal resistance between the center and the border of the n-th compartment in kΩ and C_*n*_ denotes the membrane capacity in μF. If a compartment has more than two neighboring compartments the dots must be replaced with the appropriate terms.

### 2.3 Active model

Historically, RBCs were initially assumed to be passively responding neurons. However, during the last two decades, successive new discoveries of active, voltage-gated ion channels in the membrane of RBCs have been made (e.g. Cui & Pan, 2008; Fyk-Kolodziej & Pourcho, 2007; Hu & Pan, 2002; Hu, Bi, & Pan, 2009; Ivanova & Müller, 2006; Müller et al., 2003; Pan & Hu, 2000. The ion channel complements for this article were extracted from this literature and presented in a previous study (Benav, 2012)).

For the active model the previously proposed ion channel equipment (Benav, 2012) was simplified to a model that only contains Ca^++^ T-type channels (OFF CBC) or Ca^++^ L-type channels (ON CBC) in their synaptic terminals to investigate [Ca^++^]_*i*_ which is responsible for synaptic activity.

T-type and L-type Ca^++^ currents have been found in rat CBCs. A detailed study on the differential expression of T-type and L-type Ca^++^ channels proposed that rat CBCs could be divided into two groups: T-rich cells with prominent T-type (probably somatic) and weaker L-type (probably in synaptic terminals) Ca^++^ currents or L-rich cells with more L-type (probably somatic) and less T-type (probably in synaptic terminals) Ca^++^ currents (Hu et al., 2009). Strong T-type Ca^++^ currents have been found previously in rat type 5 and type 6 CBCs (Ivanova & Müller, 2006), which are ON cells. To maximize the differences between the ON and OFF, the ON model was therefore set to be a T-rich cell (i.e. L-type channels in the synaptic terminals) while the OFF model was L-rich (i.e. T-type channels in the synaptic terminals).

The conductance-based calculation of ionic currents in dependence of V_*m*_ was performed in a custom made multi-compartment neuronal membrane model using Mathworks Matlab. A set of differential equations was used for each ion channel type, based on the formalism developed by Hodgkin and Huxley (Hodgkin & Huxley, 1952).

#### 2.3.1 Ca^++^ current models

The Nernst-potential of Ca^++^ depended on [Ca^++^]_*i*_ and was calculated dynamically. However, due to extremely low [Ca^++^]_*i*_ the majority of outward current through Ca^++^ channels is carried by K^+^ ions (Lee & Tsien, 1982). Therefore, the effective equilibrium potential of Ca^++^ channels is influenced by the K^+^ equilibrium potential. Different ratios were used for the T-type and L-type Ca^++^ channel models.

A model developed previously for T-type Ca^++^ currents in HEK-293 cells (Human Embryonic Kidney Cells) transiently expressing human Cav3.3 channel subunits (Traboulsie et al., 2007) was adapted for simulation of T-type Ca^++^ currents in rat CBCs with one activating gate and one inactivating gate. The model appropriately reproduced experimentally measures rat CBC T-type Ca^++^ currents (Hu et al., 2009).

The T-type channel was implemented in the following manner:

(3) $$i_{\mathrm{CaT}}={\overset{¯}{g}}_{\mathrm{CaT}}\mathrm{mh}(V_{m}-V_{\mathrm{CaT}})$$

with ${\overset{¯}{g}}_{\mathrm{CaT}}$=0.954mS/cm^2^ and

(4) $$\frac{\mathrm{dm}}{\mathrm{dt}}=\frac{m_{\infty }-m}{\tau_{m}}\;\frac{\mathrm{dh}}{\mathrm{dt}}=\frac{h_{\infty }-h}{\tau_{h}}$$

and

(5) $$m_{\infty }=\frac{1}{1+\exp(-(V_{m}-37.55)/3.07)}\tau_{m}=1.36+\frac{21.68}{1+\exp((V_{m}-39.96)/4.11)}\mathrm{ms}$$

(6) $$h_{\infty }=\frac{1}{1+\exp((V_{m}-8.97)/8.42)}\tau_{h}=65.82+0.0023\exp((V_{m}-80)/4.78)\mathrm{ms}$$

**Fig. 3:**
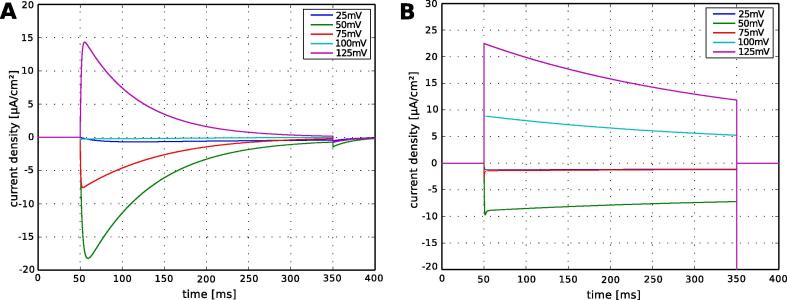
Current densities for CaT and CaL-type during voltage clamp mode – (A) Current density over time for one synaptic compartment of the OFF CBC with T-type calcium channels during voltage clamp simulations. Holding voltage was set to −80 mV, pulses of 25, 50, 75, 100 and 125 mV were applied. (B) The same simulations were conducted for the ON CBC with L-type channels. Holding voltage was −70 mV and pulses to −45, −20, +5, +30 and  + 55 mV were delivered for 300 ms.

Fig. 3A shows the current density over time for one synaptic compartment during simulated voltage clamp experiments. The holding potential was −80 mV and was increased by increments of 25 mV up to 45 mV.

Two different models were combined to optimally simulate voltage-dependent activation and inactivation of L-type Ca^++^ currents in rat CBCs. The two activation gates were adapted from a model developed for L-type Ca^++^ currents in feline RGCs (Benison, Keizer, Chalupa, & Robinson, 2001), while the inactivation gate was based on a model created for rat hippocampal CA3 pyramidal neurons (Avery & Johnston, 1996).

Similar to the T-type channel, the L-type channel was implemented with two activating gates and one inactivating gate:

(7) $$i_{\mathrm{CaL}}={\overset{¯}{g}}_{\mathrm{CaL}}m^{2}h(V_{m}-V_{\mathrm{CaL}})$$

with ${\overset{¯}{g}}_{\mathrm{CaL}}$=1.088 mS/cm^2^ and

(8) $$\frac{\mathrm{dm}}{\mathrm{dt}}=\alpha_{m}(1-m)-\beta_{m}m\;\frac{\mathrm{dh}}{\mathrm{dt}}=\frac{h_{\infty }-h}{\tau_{h}}$$

and

(9) $$\alpha_{m}=0.427\frac{V_{m}-63}{1-\exp(-(V_{m}-63)/10.5)}\;\beta_{m}=0.0406\exp((70-V_{m})/12)$$

(10) $$h_{\infty }=\frac{1}{1+\exp(V_{m}/66.4)}\;\tau_{h}=292\mathrm{ms}$$

Fig. 3B shows the current density for one synaptic terminal compartment during a simulated voltage clamp procedure. Holding potential was set to −70 mV and clamp voltages were −45, −20, +5, +30 and  + 55 mV.

${\overset{¯}{g}}_{\mathrm{CaT}}$ and ${\overset{¯}{g}}_{\mathrm{CaL}}$ had to be modified to match the data from a previous study (Benav, 2012) since the current study uses slightly different ON and OFF cell morphologies.

#### 2.3.2 Ca^++^ concentration model

The intracellular Ca^++^ concentration [Ca^++^]_*i*_ was – in contrast to all other ion concentrations – not treated as a constant and modeled in dependence of Ca^++^ currents, based on a model previously used for retinal ganglion cells (Fohlmeister, Coleman, & Miller, 1990; Rattay & Resatz, 2004):

(11) $$\frac{d{\left[{\mathrm{Ca}}^{++}\right]}_{i}}{\mathrm{dt}}=-\frac{-{\mathrm{si}}_{\mathrm{Ca}}}{2\mathrm{vF}}-\frac{{\left[{\mathrm{Ca}}^{++}\right]}_{i}-{\left[{\mathrm{Ca}}^{++}\right]}_{\mathrm{res}}}{\tau }$$

where s/v is the compartments ratio of surface to volume, F is the Faraday constant (96489C/M), 2 is the factor for Ca^++^ valence, [Ca^++^]_*res*_ is the minimal residual Ca^++^ concentration level (0.15 μM), and τ is the time constant (1.5 ms). The initial value for [Ca^++^]_*i*_ at time  = 0 was 0.1503 μM in the ON-model and 0.1501 μM in the OFF-model, slightly above the minimal level. Extracellular Ca^++^ concentration [Ca^++^]_*o*_ which was necessary to calculate the Nernst potential of Ca^++^ was not modeled and kept constant at 1800 μM, the temperature was set to the actual experiments temperature (25° C).

#### 2.3.3 Leak current model

Furthermore, a passive, linear leak current model was included. $E_{\mathrm{leak}}$ was set to −53.08 mV, the conductivity was set to $g_{\mathrm{leak}}$ = 0.0417 mS/cm^2^. The resting membrane potential ($V_{\mathrm{rest}}$) was equal to $E_{\mathrm{leak}}$. A specific membrane capacitance ($c_{m}$) of 1.1 μF/cm^2^ and a cytoplasmatic axial resistivity ($\rho_{a}$) of 130Ωcm were assumed (Oltedal, Veruki, & Hartveit, 2009). When simulations were conducted in passive mode, ${\overset{¯}{g}}_{\mathrm{CaT}}$ and ${\overset{¯}{g}}_{\mathrm{CaL}}$ were set to 0.

### 2.4 Morphological models

**Fig. 4:**
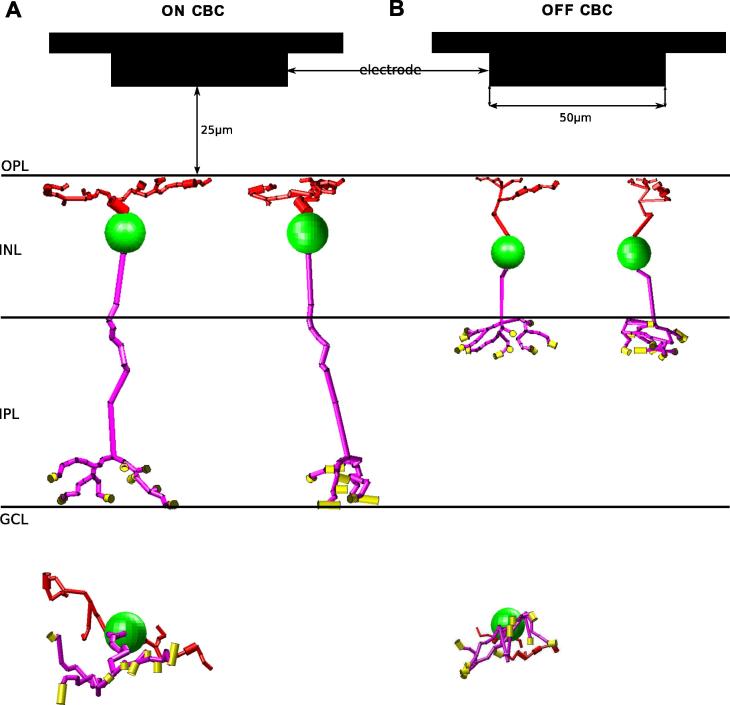
Morphological 3D models of traced rat CBCs and stimulation configuration – The images are based on 2D images (Euler & Wässle, 1995) and were created with a custom made Matlab program. (A) Type 9 ON CBC in frontal view (top left), lateral view (top right) and top view (bottom). (B) Type 3 OFF CBC in frontal view (top left), lateral view (top right) and top view (bottom). The stimulating electrode (50 μm in diameter) was positioned 25 μm from the dendritic tree in all simulations. Dendrites, soma, axon and terminals are indicated in red, green, purple and yellow, respectively.

Due to the lack of three-dimensional representations from traced CBC morphologies in publicly available databases, a custom-made Matlab tool was developed for creation of such morphologies (Encke, Benav, Werginz, Zrenner, & Rattay, 2013). *x*- and *z*-dimension of the morphological model can be extracted from 2D print images of traced bipolar cells. Cellular extent into the *y*-dimension (depth) is generated using a confined, normally distributed random variable based on the 2D extent of neuronal processes. For the ON model, an identified type 9 rat CBC (Euler & Wässle, 1995) was used. Projections of the ON model morphology are shown in Fig. 4A from a frontal, lateral and top position. The type 3 CBC was used for the morphology of the OFF model (Fig. 4B). The ON model neuron consisted of 94 compartments whereas the OFF geometry consisted of 78 compartments. In all simulations, the dendritic tree had a distance of 25 μm to the surface of the stimulating electrode.

### 2.5 Stimulation parameters

All stimulations were either performed in voltage clamp mode to explore the behavior of the various ion channel models or as extracellular stimulation to investigate the influence of an external electric field on the model fibers.

**Fig. 5:**
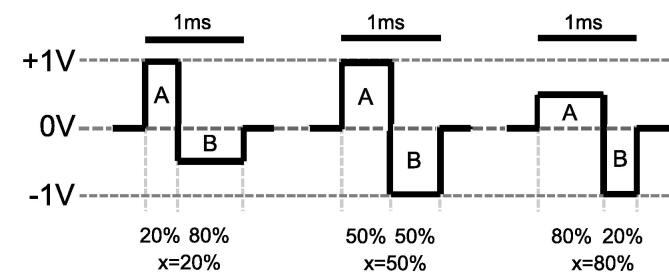
Stimulus paradigm used for the parametric investigation of biphasic anodic first pulses – The length of the first pulse determined the parameter *x*. Areas A and B were equal for each simulation. Biphasic cathodic first simulations were conducted analogously.

Monophasic rectangular anodic (hyperpolarizing) and cathodic (depolarizing) pulses were delivered as well as biphasic anodic- and cathodic-first stimuli, for the stimulating paradigm see Fig. 5. Also the response of the model neurons to stimulus bursts (consecutive pulses) and sinusoidal stimulation was explored.

Default pulse-length used in simulations was 0.5 ms or 1 ms, as these are also common in clinical trial (Zrenner et al., 2011). 1 V was set as default stimulating amplitude, since stimulation amplitudes with the Tübingen subretinal implant typically range between 0–2 V and maximally reach 2.5 V to prevent tissue damage (Sachs et al., 2005). The choice of 1 V rather than a value closer to the charge injection limit leaves additional room for further optimizations.

## 3 Results

In a first step the previously described passive model was used to determine the influence of geometric factors on the excitation of the two model neurons. Second, simulations with the active model were conducted to examine which stimulating paradigms might be able to stimulate either ON or OFF CBCs selectively.

As described previously, the key assumption of the model was that ON-type CBCs exhibit L-type calcium channels in their synaptic terminals whereas OFF-type CBCs show T-type channels. To test this hypothesis we computed the response of both cells containing the same ion channel equipment as well as we tested how the two model neurons respond to electric stimulation without any voltage gated ion channels (passive model). Furthermore, monophasic, biphasic, repetitive and sinusoidal stimulation has been tested to examine what stimulation paradigms might activate ON and OFF cells differently.

### 3.1 Influence of the cell geometry

**Fig. 6:**
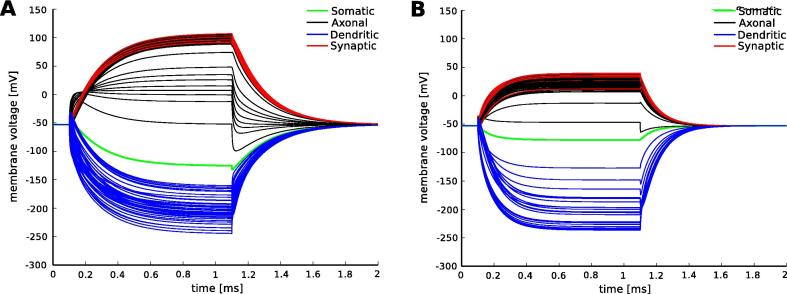
Different excitation of the two model neurons without ion channels (passive model) – Every line shows the temporal response of a single compartment during an cathodic 1 V/1 ms electrode pulse. The ON CBC (A) synaptic compartments depolarize to a higher peak membrane potential than the OFF CBC (B) whereas the dendritic compartments hyperpolarize to a similar membrane voltage in both geometries.

To determine the influence of various geometric properties of the model neurons (e.g. cell length, compartment diameter) the ON and OFF model morphologies were stimulated with a standard cathodic pulse (1 ms, 1 V) applied by a disc electrode (radius  = 25 μm). The impact of both cell shapes in the applied field is obvious (Fig. 6). The synaptic compartments of the ON CBC depolarize to a maximum transmembrane voltage of +107 mV ($\Delta V_{m}$=160 mV) whereas the dendritic compartments maximally hyperpolarize to approximately −244 mV ($\Delta V_{m}$ = 191 mV). The membrane voltage of the OFF CBC synaptic compartments increases to a maximum of +40 mV ($\Delta V_{m}$ = 93 mV) and in the dendritic compartments V_*m*_ decreases to −237 mV ($\Delta V_{m}$=184 mV).

### 3.2 Influence of axon length

**Fig. 7:**
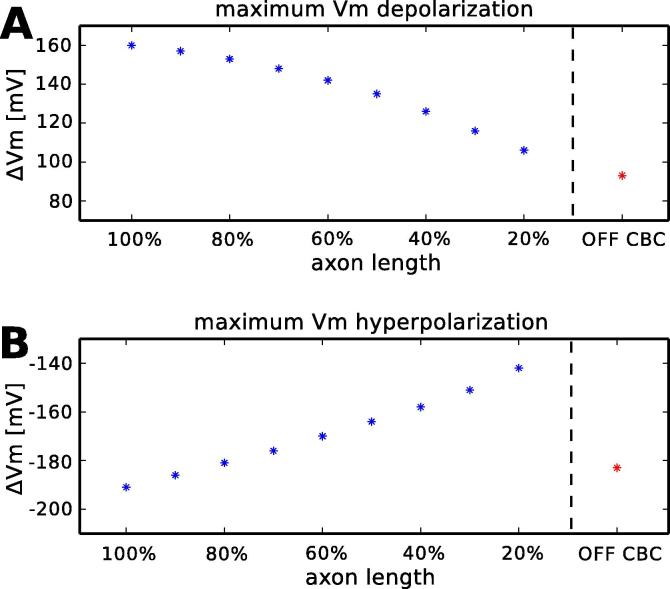
Maximum de- and hyperpolarization of the ON CBC with different axonal length – When the axon of the ON CBC is shortened, the depolarization (A) and hyperpolarization (B) become weaker. If the ON CBC has the same length as the OFF CBC (40%) the de- and hyperpolarization characteristics are totally different in both geometries.

In the next step the ON CBC axon was clinched in axial (*z*-dimension) direction in increments of 10% from 100% (original ON type geometry) to 20% of the original axial length. 40% of the original axon length results in a morphology which is comparable in length with the OFF CBC geometry, however, it shows totally different de- and hyperpolarization characteristics (Fig. 7A and B). The shortened ON type geometry depolarizes more than the OFF type geometry ($\Delta V_{m\text{,}\mathrm{ON}}$ = 126 mV, $\Delta V_{m\text{,}\mathrm{OFF}}$ = 93 mV) and hyperpolarizes less ($\Delta V_{m\text{,}\mathrm{ON}}$ = 158 mV, $\Delta V_{m\text{,}\mathrm{OFF}}$ = 184 mV).

### 3.3 Influence of compartment diameters

While a change of the axonal length leads to large differences in de- and hyperpolarization, several other geometric influence factors do not significantly affect cell activation. An increase/decrease of the soma diameter of the ON CBC results only in small differences regarding synaptic depolarization. An increase of the soma diameter by 3 μm (d = 14 μm) results in a depolarization of $\Delta V_{m}$ = 164 mV for the ON cell synaptic compartments which only differs slightly from the maximum synaptic depolarization of the standard morphology (d = 11 μm, $\Delta V_{m}$ = 160 mV). When the diameter is decreased to 8 μm $\Delta V_{m}$ drops to 156 mV. The OFF CBC also shows that the soma diameter does not affect the synaptic depolarization strongly. The standard cell geometry with a soma diameter of 9 μm results in a depolarization of $\Delta V_{m}$ = 93 mV while an increase to 12 μm leads to a $\Delta V_{m}$ of 95 mV. A smaller diameter (6 μm) leads to a maximum synaptic depolarization of $\Delta V_{m}$ = 90 mV.

Furthermore, since synaptic compartments are the crucial elements for signaling activity between RBCs and retinal ganglion cells the impact of the size of synaptic compartments diameter was tested. Larger (doubled) diameters lead to a smaller depolarization (−9% for the ON CBC and −10% for the OFF CBC) while smaller (halved) diameters lead to an increase of $\Delta V_{m}$ of +5% for the ON CBC and +6% for the OFF CBC (data not shown).

### 3.4 Effects of voltage gated Ca^++^ channels

For further investigations the passive model was extended with calcium channels in the synaptic terminals since the intracellular calcium concentration controls the synaptic activity between bipolar cells and retinal ganglion cells. A previous study (Hu et al., 2009) did not make any assumptions whether T-rich (i.e. strong T-type currents) or L-rich (i.e. strong L-type currents) cells can be divided into subgroups with different functions (i.e. ON or OFF CBCs). However, Ivanova and Müller (Ivanova & Müller, 2006) measured strong T-type currents in type 5b, 6a and 6b CBCs which are all ON type cells. Furthermore, very weak L-type currents were reported in type 3 CBCs which are OFF type cells. Therefore, L-type calcium channels were added to the ON CBC terminal compartments and T-type channels to the OFF CBC synaptic compartments, respectively (see methods ’Ca^++^ current models’).

### 3.5 Influence of axonal length on synaptic [Ca^++^]_*i*_

**Fig. 8:**
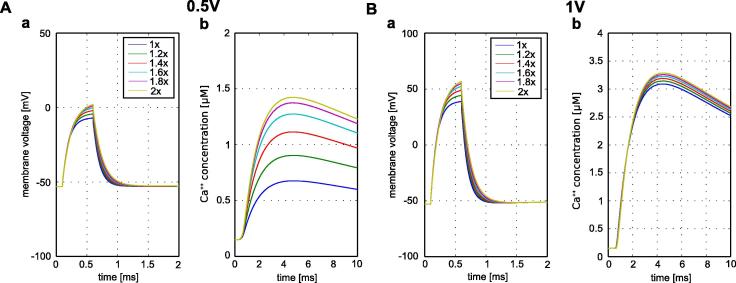
Axonal length and pulse amplitude influence synaptic [Ca^++^]_*i*_ – The OFF CBC geometry was manipulated by elongating the axonal and synaptic parts in axial direction. Multiplicators in the legend correspond to the elongation factor, i.e. ‘1x’ means the standard OFF CBC and ‘2x’ a twice as long axonal and synaptic portion. (A) A 0.5 V pulse leads to a depolarization of 43 mV (standard OFF CBC, ‘1x’) and up to 58 mV (‘2x’). The longer the cell gets, the higher increases synaptic [Ca^++^]_*i*_ with a difference of over 200% between the standard geometry and the longest version. (B) The situation changes when a 1 V pulse is applied. Again, depolarization varies between the different morphologies, however, synaptic [Ca^++^]_*i*_ only changes little.

To investigate how the intracellular calcium concentration changes when the axon length is varied we used the OFF CBC morphology as standard cell and elongated the axonal and synaptic parts. In Fig. 8 membrane voltage over time and the corresponding [Ca^++^]_*i*_ for different lengths are shown. A 0.5 V pulse (Fig. 8A) leads to small variations of V_*m*_ (panel (a)). The synaptic compartments of the standard geometry depolarize to a maximum of approximately −10 mV whereas a longer axonal and synaptic portion (in *z*-direction) results in a slightly higher membrane voltage of +5 mV. Intracellular calcium, however, shows large differences from 0.66 μM up to 1.41 μM (panel (b)).

If a stronger pulse with an amplitude of 1 V is applied, the picture changes. Although the differences in membrane voltage between the different axonal lengths remain almost the same (but on a higher level of depolarization) the intracellular calcium concentration in the synaptic compartments only varies by 7% (∼3.1–3.3 μM, Fig. 8B panel (b)). This happens likely because of a maximum inward calcium current which can not be exceeded and therefore also the internal calcium concentration is limited.

### 3.6 Identical channel equipment on both model neurons

**Fig. 9:**
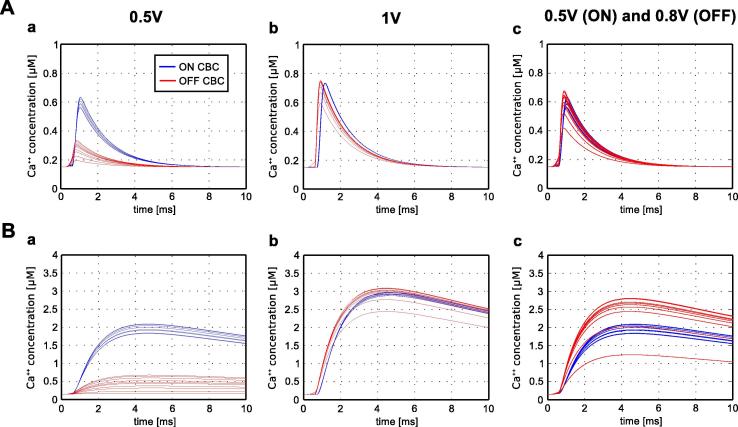
Identical Ca^++^ channels on ON and OFF morphology – If both model geometries have the same channel equipment the intracellular calcium concentration depends on how strong the neuron is depolarized. (A) Panel (a) shows [Ca^++^]_*i*_ over time for all synaptic compartments of the ON (blue traces) and OFF (red traces) CBC when the L-type channel is implemented in both geometries. The ON CBC depolarizes more than the OFF CBC during a 0.5 V pulse as also shown in Fig. 6. Intracellular calcium concentration raises more in the ON CBC as well. Although the ON CBC still gets stronger depolarized this changes when a 1 V pulse is applied (panel (b)). When both cells are depolarized similar which can be achieved by stimulating the ON CBC with a 0.5 V pulse and the OFF CBC with a 0.8 V pulse (synaptic membrane voltage increases in both cases up to  + 20 mV) synaptic calcium concentration does not differ between both cells (panel (c)). (B) If the T-type channel is implemented in both geometries and a 0.5 V pulse is applied synaptic calcium again increases higher in the ON CBC whereas a 1 V pulse leads to similar levels of [Ca^++^]_*i*_ in ON and OFF CBC. An equal depolarization (panel (c)) leads to higher calcium levels in the OFF CBC due to a faster depolarization characteristic (see Fig. 6). Pulse length was 0.5 ms in all simulations. (For interpretation of the references to color in this figure legend, the reader is referred to the web version of this article.)

An identical ion channel equipment in both cells, i.e. either L- or T-type channels in the synaptic compartments of both geometries, shows that synaptic calcium concentration is mainly influenced by the applied pulse (amplitude and length) and the depolarization characteristic of the model neuron. In Fig. 9A the L-type channel was implemented in the ON and OFF CBC. A 0.5 ms, 0.5 V pulse leads to a higher increase of [Ca^++^]_*i*_ in the ON CBC because of a more depolarized membrane (panel (a)). However, when a 1 V pulse is applied calcium increases in both geometries to the same value which is again likely because of the maximum inward calcium current and a consequent distinct maximum internal calcium concentration as can be seen in panel (b). Panel (c) shows that if both neurons are depolarized equally to about +20 mV [Ca^++^]_*i*_ also does not differ between the two neurons.

The same results were achieved when both morphologies exhibited the T-type channel. Weak pulses (<1 V) lead to a significant difference in intracellular calcium which vanish if pulse amplitude increases (Fig. 9B, panel (b)). An equal depolarization state in both neurons (+20 mV) results in a higher synaptic [Ca^++^]_*i*_ in the OFF CBC probably because of a faster depolarization.

### 3.7 Influence of the electrode position

**Fig. 10:**
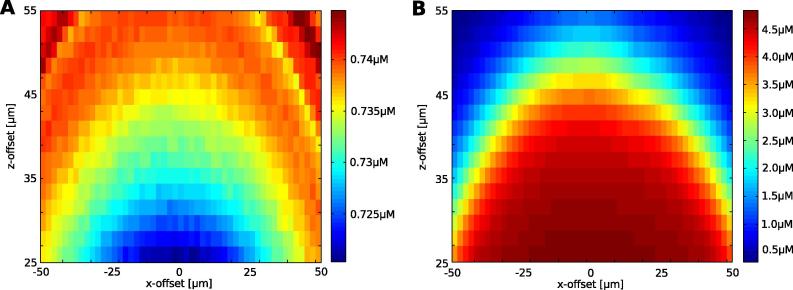
Maximum [Ca^++^]_*i*_ in synaptic terminals for different cell positions relative to the electrode – The standard position of the electrode is represented by the center pixel in the bottom row. This position corresponds to a centered electrode above the cell soma (*x*- and *y*-axis) and 25 μm distant from the dendritic tree in *z*-direction. (A) Synaptic compartments of the ON CBC increase their [Ca^++^]_*i*_ up to 0.75 μM. Interestingly, the peak calcium concentration was found to be at a position further away from the stimulating electrode than in the standard case, however, variations are quiet small. (B) Changes of synaptic [Ca^++^]_*i*_ are larger in the OFF CBC. Stimulation from the standard electrode position results in maximum [Ca^++^]_*i*_ of approximately 4.5 μM.

Fig. 10 shows the variations in maximum synaptic [Ca^++^]_*i*_ at the terminal compartments for 816 different cell positions relative to the stimulating electrode when a standard pulse (1 ms, 1 V, monophasic, cathodic) is delivered. The center pixel in the bottom row represents the standard position, the cell-soma is centered above the electrode (in *x*- and *y*- direction), the distance between the dendritic tree and the electrode is 25 μm in *z*-direction. When the electrode is shifted in *x*-direction as well as in *z*-direction the maximum synaptic calcium concentration changes. Each pixel represents a 2 μm shift. Note that the two-dimensional maps in Fig. 10 are not exactly symmetric because of the asymmetric cell morphologies.

Furthermore, the ON CBC in Fig. 10A does not have its maximum [Ca^++^]_*i*_ at the standard electrode position like the OFF CBC (Fig. 10B). For the ON CBC the change of [Ca^++^]_*i*_ is fairly small (minimum = 0.72 μM, maximum = 0.75 μM) whereas the OFF CBC shows larger variation (minimum = 0.5 μM, maximum = 4.5 μM).

### 3.8 Monophasic stimulation

**Fig. 11:**
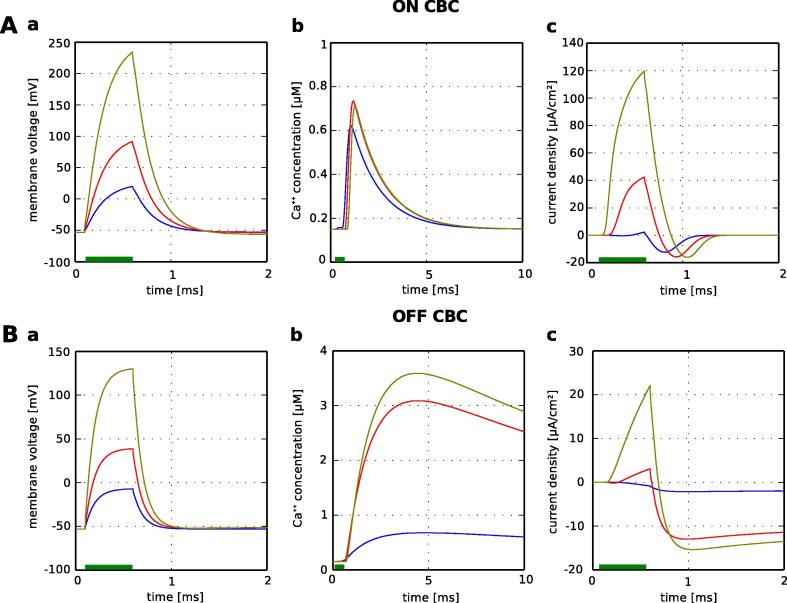
Monophasic stimulation with three different stimulus amplitudes – Three 0.5 ms cathodic pulses with amplitudes of 0.5 V (blue trace), 1 V (red trace) and 2 V (yellow trace) were applied. (A) One synaptic compartment of the ON CBC depolarizes to membrane voltages (a) of  + 21 mV, +93 mV and  + 236 mV during the three pulses, the internal calcium concentration (b) increases to 0.61 μM, 0.74 μM and 0.73 μM, respectively. Current densities of the L-type calcium channels are shown in (c). (B) The membrane voltage (a) of one synaptic terminal of the OFF CBC depolarizes to lower values than the ON CBC (-7 mV, 41 mV, +130 mV) during the three pulses. Synaptic [Ca^++^]_*i*_ (b) raises to 0.7 μM during the 0.5 V stimulus and to 3.05 μM and 3.6 μM during the stronger pulses. In (c) the current density of the calcium channel shows similar characteristics as in the ON CBC. The green bars at the bottom indicate the stimulus on- and offset.

To examine the influence of the incorporated voltage gated calcium channels, monophasic, rectangular pulses with a length of 0.5 ms were applied with different stimulus amplitudes. Fig. 11A shows membrane voltage (left), [Ca^++^]_*i*_ (middle) and calcium currents (right) of one synaptic compartment for three different pulse amplitudes on the ON (Fig. 11A) and OFF CBC (Fig. 11B). An applied voltage of 0.5 V leads to a depolarization of the ON CBC of approximately 74 mV, [Ca^++^]_*i*_ increases to a maximum of 0.61 μM and a small outward current (1 μA/cm^2^) followed by an inward current with a density of about 16 μA/cm^2^ occurs. When the stimulus amplitude increases, the synaptic compartment depolarizes more (146 mV during a 1 V pulse, 289 mV during a 2 mV pulse) and [Ca^++^]_*i*_ increases to 0.74 μM and 0.73 μM, respectively. Outward calcium currents (41 μA/cm^2^ and 120 μA/cm^2^, respectively) occur during the depolarization of the cell, smaller inward currents (∼18 μA/cm^2^) after the membrane voltage falls to resting voltage again.

The OFF CBC depolarizes less than the ON CBC during the three stimulations as shown in Fig. 11B. A 0.5 V pulse leads to a maximum membrane voltage of approximately −7 mV, a 1 V pulse to +41 mV and a 2 V pulse to +130 mV. [Ca^++^]_*i*_ only increases slightly to 0.7 μM when a 0.5 V pulse is delivered, 1 V and 2 V pulses lead to 3.05 μM and 3.6 μM, respectively. The calcium current is totally inward during the 0.5 V pulse, during the 1 V and 2 V pulses also outward current densities occur when the cell depolarizes. Inward peak amplitudes are 2 μA/cm^2^, 12 μA/cm^2^ and 15 μA/cm^2^, respectively.

Interestingly, although the ON CBC gets more depolarized than the OFF type cell during all pulses, higher calcium concentrations and more sustained calcium levels can be evoked in the OFF CBC. This discrepancy results from two major reasons: (a) the density of calcium channels in the synaptic terminals is (slightly) different in both cells and (b) the kinetics of the two channel types are not the same. The fairly fast reversion to resting state of [Ca^++^]_*i*_ in the ON CBC is caused by a de-activation (not inactivation) since the membrane voltage drops back to resting potential within 1 ms after pulse offset. Because the activation variable m is almost 0 at resting potential the synaptic calcium influx stopped immediately afterwards and returned fast (4–5 ms) back to its resting value. The time constant of the T-type channel on the other hand has larger values at resting potential than in depolarized states. Therefore, the slower change of activation also leads to a slow decrease of the calcium current amplitude and therefore to a sustained [Ca^++^]_*i*_ level.

The described behavior of the two ion channels as well as the different de- and hyperpolarization characteristics are probably the reasons for the fairly large differences in Fig. 10.

### 3.9 Biphasic stimulation

**Fig. 12:**
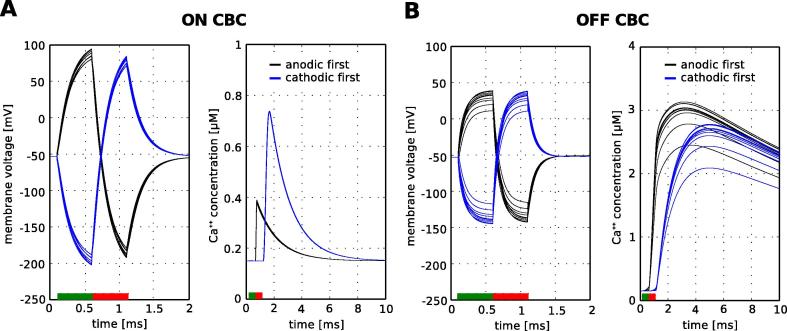
Membrane voltage over time and synaptic [Ca^++^]_*i*_ during biphasic stimulation – (A) The time course of the membrane voltage of all synaptic compartments of the ON CBC is depicted in the left panel. During the anodic-first pulse (black trace), the terminals depolarize slightly more than for the cathodic-first case (blue trace). The anodic-first pulse applied on the ON CBC leads to a peak [Ca^++^]_*i*_ of approximately 0.39 μM (right panel) whereas a cathodic-first pulse results in an intracellular calcium concentration of 0.77 μM. (B) Synaptic terminals of the OFF CBC depolarize to approximately 45 mV in both stimulating configurations. [Ca^++^]_*i*_ increases to a maximum value of 2.9 μM (cathodic-first) and 3.1 μM (anodic-first). The green and red bars at the bottom indicate the length of the stimulus as well as the switch between cathodic and anodic pulse phase.

Anodic- and cathodic-first pulses (x = 50%, see methods and Fig. 5) were applied on the two model neurons to examine how [Ca^++^]_*i*_ changes. Fig. 12A displays the time course of the transmembrane voltage during 1 V anodic-first (black trace) and cathodic-first (blue trace) pulses with a length of 1 ms overall. The synaptic compartments of the ON CBC depolarize up to approximately 90–100 mV for both stimulation modi. Resulting peak [Ca^++^]_*i*_ for the ON CBC was 0.39 μM during anodic-first stimulation and 0.77 μM during cathodic-first stimulation (see Fig. 12A). In Fig. 12B it can be seen that the membrane voltage depolarizes less than in the ON CBC to approximately 45 mV. [Ca^++^]_*i*_ in the OFF CBC synaptic compartments increases to about 3.1 μM when a anodic-first stimulus is applied and to 2.9 μM during a cathodic-first pulse. In sum, cathodic-first stimulation leads to higher [Ca^++^]_*i*_ in the ON CBC whereas for the OFF CBC anodic-first stimulation results in a higher [Ca^++^]_*i*_.

### 3.10 Repetitive pulses

Since retinal implants use stimulus bursts to evoke visual perceptions in blind people it was tested how repetitive stimulation would affect synaptic [Ca^++^]_*i*_ in the two model neurons. A monophasic, cathodic pulse with a pulse length of 0.5 ms and a stimulus amplitude of 1 V was applied 5 times. Inter-stimulus intervals between the 5 consecutive pulses varied between 0.5 ms and 49.5 ms.

**Fig. 13:**
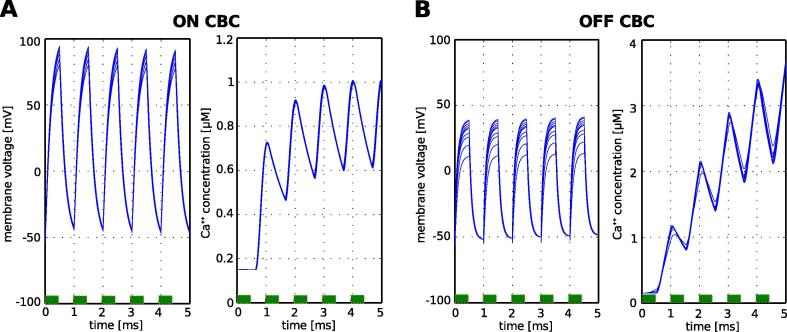
Repetitive stimulation results in different [Ca^++^]_*i*_ in ON and OFF CBC terminals – (A) The ON CBC responds to 5 consecutive monophasic cathodic pulses (0.5 ms pulse length, 0.5 ms inter stimulus interval, 1 V) with a peak [Ca^++^]_*i*_ of approximately 1 μM. (B) Because of persistent calcium T-type currents, the calcium concentration in the synaptic compartments raises up to 3.8 μM during repetitive stimulation in the OFF CBC. Again, the green bars at the bottom indicate pulse on- and offset.

A 1 ms stimulus period (0.5 ms pulse  + 0.5 ms inter-stimulus interval) results in slightly increasing [Ca^++^]_*i*_ during consequent pulses (Fig. 13A). The first pulses raises [Ca^++^]_*i*_ to approximately 0.75 μM, the following pulses increase [Ca^++^]_*i*_ in the synaptic compartments to a peak value of 1 μM. After pulse offset, [Ca^++^]_*i*_ went back to its resting state (0.1503 μM) within 4–5 ms (data not shown).

When the same stimulation paradigm is applied to the OFF CBC [Ca^++^]_*i*_ increases to values up to 3.8 μM after the last of the 5 single pulses (Fig. 13B). After the last pulse it takes about 40–50 ms to bring back [Ca^++^]_*i*_ to its initial value of 0.1501 μM.

### 3.11 Sinusoidal pulses

To investigate the influence of sinusoidal stimulation on synaptic calcium currents we simulated how three different ion channels respond to sinusoidal stimulation with different frequencies. The previously presented calcium L- and T-type calcium channels as well as a sodium channel were investigated in a mono-compartment model. Sodium channel kinetics were taken from Benison and coworkers (Benison et al., 2001). The additional sodium channel was incorporated to investigate which parts of the retina might be preferably activated with sinusoidal stimulation (see Discussion).

**Fig. 14:**
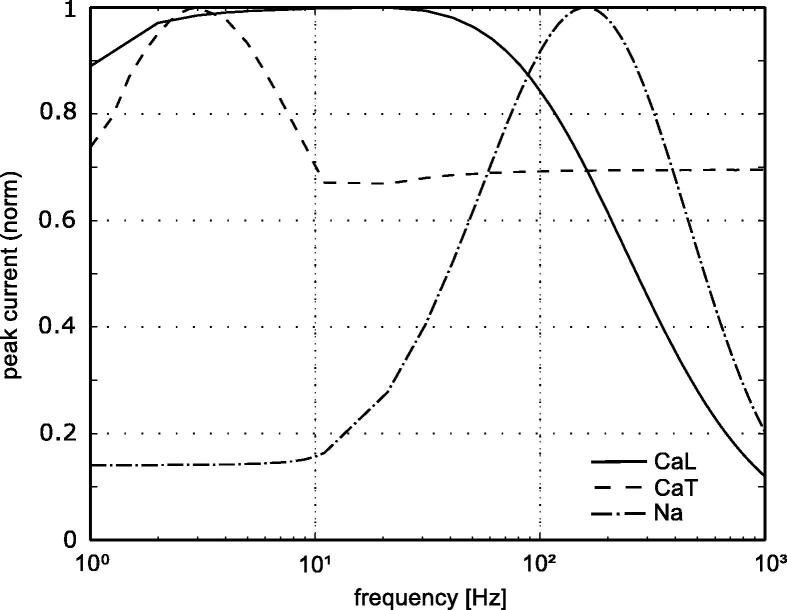
Ion channel activation is stimulus frequency-dependent – A mono-compartment model was established with three voltage gated ion channels to investigate how those are activated during sinusoidal stimulation. Calcium L-type (solid trace), calcium T-type (dashed trace) and a sodium channel (dashed-dotted trace) from a previous study (Benison et al., 2001) were tested. The calcium T-type channel activates strongly at frequencies around 3–5 Hz and is still open at high frequencies. L-type currents peak at slightly higher frequencies, however, the channel closes when frequency increases. Maximum sodium current is evoked by stimulation around 200 Hz.

Stimulus frequency was varied from 1 up to 1000 Hz and the evoked ionic currents were normalized. As can be seen in Fig. 14 the T-type channel (dashed line) shows its maximum activation at approximately 3 Hz and acts as a bandpass filter. L-type calcium currents (solid line) are maximal at frequencies between 8 and 20 Hz and decrease with higher frequencies. The sodium channel (dashed-dotted line) activates moderate at low frequencies and has its peak current at ∼200 Hz.

## 4 Discussion

This study presents various computer simulations trying to resemble actual physiologic processes in retinal bipolar cells during subretinal stimulation. Results from electrophysiologic recordings were investigated and a computational model which was developed previously (Benav, 2012) was modified. Furthermore, one model ON CBC and OFF CBC were reconstructed by using a previously presented tool (Encke et al., 2013). Monophasic, biphasic, single and repetitive pulses in different lengths and amplitudes were applied in passive (without ion channels) and active mode to see how the two model neurons respond to external electric stimulation.

### 4.1 Passive model

To find crucial geometric parameters in the model several simulations in a passive model were conducted. While many parameters as soma diameter and the diameter of the synaptic compartments do not have a large influence, the length of the whole fiber does. Since the ON CBC was longer than the OFF CBC it was depolarized more during the stimulation with the same anodic pulses. When the axonal length was shortened consecutively in axial-direction the depolarization characteristics became similar for both cells. However, if both cells are the same in length (40% of the original ON CBC axon length) the OFF CBC is still less depolarized which means that also other factors than length (e.g. soma position) play a role for the magnitude of depolarization.

Since the ON CBC depolarizes slower but stronger than the OFF CBC this behavior changes for very short pulse lengths (<0.2 ms) which are, however, not common in retinal implants. The reasons for the different depolarization characteristics of the synaptic compartments between ON and OFF CBCs seem to be (i) the geometry of the synaptic terminal region and (ii) the voltage gradient along the cells *z*-axis which changes for locations further away from the stimulating electrode.

### 4.2 Synaptic calcium channels

This study assumes that ON CBCs exhibit calcium L-type channels in their synaptic terminals whereas OFF CBCs show T-type channels in their synaptic compartments as proposed in a previous study (Hu et al., 2009).

During the applied pulses, the synaptic calcium concentrations reached peak values up to 1 μM for the ON CBC and up to 3.6 μM for the OFF CBC which is sufficient to initiate synaptic activity from RBCs to retinal ganglion cells (Baden, Euler, Weckström, & Lagnado, 2013; Zhou, Wan, Thakur, & Heidelberger, 2006). However, in this study no retinal network activity was investigated, other retinal neurons as amacrine cells may also have a significant influence on the signaling cascade between RBCs and retinal ganglion cells when stimulated electrically.

Identical ion channel equipment in both model neurons and elongating the axonal and synaptic portions of the OFF CBC showed that the state of depolarization in the synaptic compartments and therefore again the geometric factors (mainly length) have the greatest influence on [Ca^++^]_*i*_ during stimulation.

### 4.3 1 V and 2 V pulses result in similar [Ca^++^]_*i*_ in the ON CBC

As can be seen in Fig. 11A synaptic calcium currents (current densities) are stronger in outward (positive) than in inward (negative) direction. The two outward amplitudes for 1 V and 2 V pulses are different (41 μA/cm^2^ and 120 μA/cm^2^), however, [Ca^++^]_*i*_ shows the same time course and maximum (0.77 μM). Because the initial value for [Ca^++^]_*i*_ was close to the residual value (0.1503 μM and 0.1501 μM, respectively) the initial outward currents did not affect [Ca^++^]_*i*_ strongly. Following inward currents, however, had the same amplitudes and therefore [Ca^++^]_*i*_ did not differ between the two distinct stimulating amplitudes.

### 4.4 Direct and indirect activation of ganglion cells

When retinal implants, placed either at the inside or outside of the retina, stimulate inner eye neurons electrically two different activation mechanisms can be found: (a) direct stimulation which is triggered by the stimulating pulse itself and (b) indirect (synaptic) stimulation through cell-to-cell interaction.

Previous experimental studies reported differential activation of ON and OFF retinal ganglion cells (i.e. Jensen & Rizzo, 2006). The presented model only examines the response of bipolar cells to electrical stimulation without taking into account any network activity. Anodal monophasic stimulation using subretinal electrodes results in an increased synaptic calcium concentration and therefore might be able to indirectly activate ganglion cells via synaptic activity. Cathodal stimulation on the other hand does not lead to an increase of the internal calcium concentration, and therefore is unlikely to mediate any synaptic activation.

A possible explanation for the differences between the presented model and experimental findings might be the fact that only bipolar cells without connecting amacrine and ganglion cells have been examined. The main axes of bipolar cells are aligned perpendicular to the stimulating electrode and therefore will be activated when the first derivative of the applied voltage is positive (i.e. during anodal stimulation). However, amacrine cells and ganglion cells, which are aligned parallel to the stimulating electrode are activated in regions where the second derivative of the external potentials is positive. This concept of the activating function has been presented and discussed previously (Rattay, 1999). Therefore, using the presented model and two distinct model geometries, an anodal stimulating pulse can not lead to network (indirect) activation which might be the case in actual experiments. However, morphologies which show specific terminal geometries (e.g. terminals are aligned parallel to the stimulating element) as well as amacrine cells might also be activated during simulated anodal stimulation.

Thus, it is likely that Jensen and Rizzo (Jensen & Rizzo, 2006) activated bipolar cells as well as other parts in the retinal network and thus recorded network activation which can not be explained in this study.

Therefore, a comparison between experimental data of retinal ganglion cells and the presented modeling results is not possible without extending the model. Combining a bipolar cell, an amacrine cell, a ribbon synapse and a subsequent ganglion cell will give further insights into the mechanisms of direct and indirect stimulation.

### 4.5 Selective stimulation of ON and OFF CBCs

One of the main goals in the development of retinal implants is to avoid the co-activation of the ON and OFF pathway during stimulation. Biphasic pulses are on the one hand an opportunity to avoid charge injection into the tissue and on the other hand such pulse configurations might be able to focally activate either ON or OFF CBCs. It was shown (Fig. 12) that [Ca^++^]_*i*_ in the synaptic terminals in the ON CBC is approximately 2 times higher during a cathodic-first pulse than during an anodic-first pulse. The OFF CBC on the other hand shows a slightly stronger increase of [Ca^++^]_*i*_ when an anodic-first pulse is applied. In both cases calcium currents were initiated during the anodic phase. Therefore, it might be possible to activate either the ON or OFF pathway by using either anodic- or cathodic-first voltage pulses.

### 4.6 Repetitive and sinusoidal stimulation

The outcome of clinical studies using repetitive stimulation shows various drawbacks. The most reported disturbance of patients is the fading of visual sensations when the stimulating frequency exceeds levels of 10 Hz (Zrenner et al., 2011). Jensen and coworkers (Jensen & Rizzo, 2007) suggested that vesicle depletion might be the cause for this phenomenon. In this study, synaptic [Ca^++^]_*i*_ returned to its resting state after 4–5 ms (ON CBC) and approximately 40–50 ms (OFF CBC), however, repetitive stimulation was not shown to be sustainable under these conditions. Therefore, there may be other limiting factors that underlie the fading of percepts during clinical application.

Since previous studies (Freeman, Eddington, Rizzo, & Fried, 2010; Freeman, Jeng, Kelly, Hartveit, & Fried, 2011) reported that sinusoidal waveforms with different frequencies might be able to differentially activate bipolar and ganglion cells, respectively, we also examined how such pulses activate the presented ion channels. We resembled Fig. 7G from Freeman and coworkers (Freeman et al., 2010) to see if the presented ion channels support their assumptions (Fig. 14). Our L- and T-type channels show similar characteristics during sinusoidal stimulation. Therefore, as stated before, lower frequencies in fact might activate bipolar cells and photoreceptors stronger whereas higher frequencies activate sodium channels and thus ganglion cells preferably.

### 4.7 Limitations

Modeling and simulation of neuronal tissue during external electric stimulation exhibits several limitations. As mentioned before, this study only investigates the activation of bipolar cells without taking into account other neuron-to-neuron connections in the complex retinal network. Therefore, other neurons as amacrine cells can have a substantial influence on the synaptic activity between bipolar cells and ganglion cells. Furthermore, to differentiate between ON and OFF CBCs, the ON CBC model was chosen to be T-rich whereas the OFF CBC model was L-rich (Hu et al., 2009) which might be not the case.

Moreover, the resistivity of the retina was presented in several studies (Karwoski, Frambach, & Proenza, 1985; Greenberg, Velte, Humayun, Scarlatis, & de Juan, 1999; Kasi, Hasenkamp, Cosendai, Bertsch, & Renaud, 2011), however, the reported values vary by a factor of 100. In this study 57 Ωcm was used which might be smaller than the actual retina resistivity. A too small resistivity, however, does not substantially influence the distribution of the electric potential in the modeled retina as calculations with higher retina resistivity have shown (results not shown).

Note that many parameters as conductivities or ion channel densities have to be estimated and therefore the results have to be regarded with care. Channel conductivities ${\overset{¯}{g}}_{\mathrm{CaL}}$ and ${\overset{¯}{g}}_{\mathrm{CaT}}$, however, only have an effect on the amplitude of the generated calcium currents and do not influence stimulation selectivity. Therefore, although the relative conductivity of the T- and L-type channel might not be estimated correctly, different values will affect magnitude of intracellular calcium but not the general conclusions of this study.

## References

[b0005] Avery R.B., Johnston D. (Sep 1996). Multiple channel types contribute to the low-voltage-activated calcium current in hippocampal ca3 pyramidal neurons. Journal of Neuroscience.

[b0010] Baden T., Euler T., Weckström M., Lagnado L. (Aug 2013). Spikes and ribbon synapses in early vision. Trends in Neuroscience.

[b0015] Benav, H., (2012). *Modelling effects of extracellular stimulation on retinal bipolar cells* (Ph.D. thesis). Eberhard-Karls-Universitaet Tuebingen.

[b0020] Benison G., Keizer J., Chalupa L.M., Robinson D.W. (May 2001). Modeling temporal behavior of postnatal cat retinal ganglion cells. Journal of Theoretical Biology.

[b0025] Cai C., Ren Q., Desai N.J., Rizzo J.F., Fried S.I. (Jul 2011). Response variability to high rates of electric stimulation in retinal ganglion cells. Journal of Neurophysiology.

[b0030] Cui J., Pan Z.-H. (2008). Two types of cone bipolar cells express voltage-gated na+ channels in the rat retina. Visual Neuroscience.

[b0035] Encke J., Benav H., Werginz P., Zrenner E., Rattay F. (2013). Investigating the influence of 3d cell morphology on neural response during electrical stimulation. Biomedizinische Technik.

[b0040] Euler T., Wässle H. (Oct 1995). Immunocytochemical identification of cone bipolar cells in the rat retina. The Journal of Comparative Neurology.

[b0045] Fohlmeister J.F., Coleman P.A., Miller R.F. (Mar 1990). Modeling the repetitive firing of retinal ganglion cells. Brain Research.

[b0050] Freeman D.K., Eddington D.K., Rizzo J.F., Fried S.I. (Nov 2010). Selective activation of neuronal targets with sinusoidal electric stimulation. Journal of Neurophysiology.

[b0055] Freeman D.K., Jeng J.S., Kelly S.K., Hartveit E., Fried S.I. (Aug 2011). Calcium channel dynamics limit synaptic release in response to prosthetic stimulation with sinusoidal waveforms. Journal of Neural Engineering.

[b0060] Fyk-Kolodziej B., Pourcho R.G. (Apr 2007). Differential distribution of hyperpolarization-activated and cyclic nucleotide-gated channels in cone bipolar cells of the rat retina. The Journal of Comparative Neurology.

[b0065] Gerhardt, M., (2010). *Verbesserung der orts- und zellselektivität der multilokalen elektrostimulation durch raumzeitliche feldmodulation* (Ph.D. thesis). Universitaet Ulm.

[b0070] Greenberg R.J., Velte T., Humayun M., Scarlatis G., de Juan E. (1999). A computational model of electrical stimulation of the retinal ganglion cell. IEEE Transactions on Biomedical Engineering.

[b0075] Hodgkin A.L., Huxley A.F. (Aug 1952). A quantitative description of membrane current and its application to conduction and excitation in nerve. The Journal of Physiology.

[b0080] Hu C., Bi A., Pan Z.-H. (2009). Differential expression of three t-type calcium channels in retinal bipolar cells in rats. Visual Neuroscience.

[b0085] Hu H.-J., Pan Z.-H. (2002). Differential expression of k+ currents in mammalian retinal bipolar cells. Visual Neuroscience.

[b0090] Ivanova E., Müller F. (2006). Retinal bipolar cell types differ in their inventory of ion channels. Visual Neuroscience.

[b0095] Jensen R.J., Rizzo J.F. (Aug 2006). Thresholds for activation of rabbit retinal ganglion cells with a subretinal electrode. Experimental Eye Research.

[b0100] Jensen R.J., Rizzo J.F. (Mar 2007). Responses of ganglion cells to repetitive electrical stimulation of the retina. Journal of Neural Engineering.

[b0105] Kameneva T., Meffin H., Burkitt A.N. (2010). Differential stimulation of on and off retinal ganglion cells: a modeling study. Conference proceedings IEEE Engineering in Medicine and Biology Society.

[b0110] Karwoski C.J., Frambach D.A., Proenza L.M. (Dec 1985). Laminar profile of resistivity in frog retina. Journal of Neurophysiology.

[b0115] Kasi H., Hasenkamp W., Cosendai G., Bertsch A., Renaud P. (2011). Simulation of epiretinal prostheses – evaluation of geometrical factors affecting stimulation thresholds. Journal of Neuroengineering and Rehabilitation.

[b0120] Lee K.S., Tsien R.W. (Jun 1982). Reversal of current through calcium channels in dialysed single heart cells. Nature.

[b0125] Müller F., Scholten A., Ivanova E., Haverkamp S., Kremmer E., Kaupp B. (2003). Hcn channels are expressed differentially in retinal bipolar cells and concentrated at synaptic terminals. European Journal of Neuroscience.

[b0130] Oltedal L., Veruki M.L., Hartveit E. (Feb 2009). Passive membrane properties and electrotonic signal processing in retinal rod bipolar cells. The Journal of Physiology.

[b0135] Pan Z.H., Hu H.J. (Nov 2000). Voltage-dependent na(+) currents in mammalian retinal cone bipolar cells. Journal of Neurophysiology.

[b0140] Rattay F. (Mar 1999). The basic mechanism for the electrical stimulation of the nervous system. Neuroscience.

[b0145] Rattay F., Resatz S. (Sep 2004). Effective electrode configuration for selective stimulation with inner eye prostheses. IEEE Transactions on Biomedical Engineering.

[b0150] Rattay F., Resatz S., Lutter P., Minassian K., Jilge B., Dimitrijevic M.R. (Jan 2003). Mechanisms of electrical stimulation with neural prostheses. Neuromodulation.

[b0155] Resatz, S., (2005). *Analysis of retinal cell excitation with visual neuroprostheses* (Ph.D. thesis). Vienna: University of Technology.

[b0160] Sachs H.G., Schanze T., Brunner U., Sailer H., Wiesenack C. (Mar 2005). Transscleral implantation and neurophysiological testing of subretinal polyimide film electrodes in the domestic pig in visual prosthesis development. Journal of Neural Engineering.

[b0165] Traboulsie A., Chemin J., Chevalier M., Quignard J.-F., Nargeot J., Lory P. (Jan 2007). Subunit-specific modulation of t-type calcium channels by zinc. The Journal of Physiology.

[b0170] Twyford P., Cai C., Fried S. (Apr 2014). Differential responses to high-frequency electrical stimulation in on and off retinal ganglion cells. Journal of Neural Engineering.

[b0175] Usui S., Ishihara A., Kamiyama Y., Ishii H. (Dec 1996). Ionic current model of bipolar cells in the lower vertebrate retina. Vision Research.

[b0180] Wässle H. (Oct 2004). Parallel processing in the mammalian retina. Nature Reviews Neuroscience.

[b0185] Zhou Z.-Y., Wan Q.-F., Thakur P., Heidelberger R. (Nov 2006). Capacitance measurements in the mouse rod bipolar cell identify a pool of releasable synaptic vesicles. Journal of Neurophysiology.

[b0190] Zrenner E., Bartz-Schmidt K.U., Benav H., Besch D., Bruckmann A., Gabel V.-P. (May 2011). Subretinal electronic chips allow blind patients to read letters and combine them to words. Proceedings of the Royal Society B-Biological sciences.

